# Extra Uterine Fœtation

**Published:** 1845-09

**Authors:** 


*Extra Uterine Fœtation.*—Dr. Joseph Edmundson, of Carrick-on-Suir, has sent us the following case:—On Tuesday evening last, at ten o'clock, I was requested to visit a poor woman, named Kuvan, who had been in labour since four o'clock on Sunday morning (sixty-six hours.)

She stated to me that this was her fifth pregnancy, that the full term of gestation had expired, but that she never had experienced such severe labour in any of her former confinements. Her countenance was expressive of great anxiety ; extremities cold ; tongue white and dry ; pulse 130 ; very weak ; abdominal tenderness and great prostration of strength ; no discharge of liquor amnii ; vagina cool and moist ; os uteri dilated to about the size of a crown piece. I thought I felt a head presentation ; not having passed any water for several hours, I introduced the catheter, and drew off about ℥xij. of turbid fœtid urine.

I then left her, having directed her to use some stimulants, and to have heated bricks immediately applied to the feet.

Two o'clock.—Pulse 125 ; somewhat stronger ; abdominal tenderness increased, particularly at the left side ; os uteri in the same state ; but I could not detect any presentation ; slight hæmorrhage, about ℥iij, of blood, or perhaps a little more. On inquiry I was told that she had had one short violent pain, and that the uterus had not acted since ; that the pain was succeeded by vomiting. I now looked upon it as a case of ruptured uterus ; the prostration of strength was such that I did not feel myself justified in attempting to turn ; I administered a small dose of tinct. opii and spt. ammon. aromat. to check the vomiting, and ordered the stimulants to be continued.

I visited her every two hours, until one o'clock, P. M. Wednesday; the pulse was then a little firmer, but no other change had taken place ; I determined to give her a chance, and make an attempt at turning the child. There had been no return of hæmorrhage ; the vagina rather hot, but still moist and dilatable. Without much difficulty I introduced my fingers and thumb (as far as the metacarpus) into the uterus, but could not succeed further ; the uterus was acting powerfully, notwithstanding my having given a dose of tinct. opii previously to. and having repeated it whilst attempting to turn. I was obliged to withdraw my hand, and desist from any further attempts ; she sank rapidly, and died at half-past six o'clock the same evening.

I was requested at nine o'clock (two and a half hours after death) to remove the child, for the purpose of having it buried separately.

Having made an oblique incision in the left side of the abdomen, I discovered an extra uterine fœtation. A fine full-grown female child was lying on the left side, between the intestines and the peritoneal lining of the abdominal parietes, the head being placed in the left iliac region. The infant was perfectly formed, and of the usual size, having lived to the full period. It had not undergone the slightest degree of decomposition, but the face was swollen and greatly contused.

The left Fallopian tube was ruptured to the extent of about two and a half inches ; the uterus was greatly hypertrophied, being about the size of a fœtal head of the sixth month; the cavity in the uterus was about four inches long, and contained some clotted blood; the placenta appeared to be about the usual size.

From the time I had seen her, I could not detect any stethoscopic signs of fœtal life, and the mother told me that Monday was the last time she was sensible of the existence of the child.—*Lond. Med. Times.*

